# Simultaneous determination of HCV genotype and NS5B resistance associated substitutions using dried serum spots from São Paulo state, Brazil

**DOI:** 10.1099/acmi.0.000326

**Published:** 2022-03-02

**Authors:** Kazeem Adeboyejo, Victória Riquena Grosche, Diego Pandeló José, Giulia Magalhães Ferreira, Jacqueline Farinha Shimizu, Barnabas J. King, Alexander W. Tarr, Márcia Maria Costa Nunes Soares, Jonathan K. Ball, C. Patrick McClure, Ana Carolina Gomes Jardim

**Affiliations:** ^1^​ NIHR Nottingham Biomedical Research Centre, Nottingham University Hospitals NHS Trust and the University of Nottingham, Nottingham, UK; ^2^​ School of Life Sciences, University of Nottingham, Nottingham, UK; ^3^​ Institute of Biomedical Sciences, Federal University of Uberlândia, Uberlândia, Minas Gerais, Brazil; ^4^​ Institute of Bioscience, Language and Exact Sciences, São Paulo State University, São José do Rio Preto, São Paulo, Brazil; ^5^​ Federal University of Triângulo Mineiro, Iturama, Minas Gerais, Brazil; ^6^​ MRC/EPSRC Nottingham Molecular Pathology Node, University of Nottingham, Nottingham, UK; ^7^​ Instituto Adolfo Lutz, São José do Rio Preto, São Paulo, Brazil

**Keywords:** direct-action antivirals, dried serum spot, genotyping, Hepatitis C Virus, NS5B, resistance-associated substitutions

## Abstract

Hepatitis C virus (HCV) is responsible for more than 180 million infections worldwide, and about 80 % of infections are reported in Low and Middle-income countries (LMICs). Therapy is based on the administration of interferon (INF), ribavirin (RBV) or more recently Direct-Acting Antivirals (DAAs). However, amino acid substitutions associated with resistance (RAS) have been extensively described and can contribute to treatment failure, and diagnosis of RAS requires considerable infrastructure, not always locally available. Dried serum spots (DSS) sampling is an alternative specimen collection method, which embeds drops of serum onto filter paper to be transported by posting to a centralized laboratory. Here, we assessed feasibility of genotypic analysis of HCV from DSS in a cohort of 80 patients from São Paulo state Brazil. HCV RNA was detected on DSS specimens in 83 % of samples of HCV infected patients. HCV genotypes 1a, 1b, 2a, 2c and 3a were determined using the sequence of the palm domain of NS5B region, and RAS C316N/Y, Q309R and V321I were identified in HCV 1b samples. Concerning therapy outcome, 75 % of the patients who used INF +RBV as a previous protocol of treatment did not respond to DAAs, and 25 % were end-of-treatment responders. It suggests that therapy with INF plus RBV may contribute for non-response to a second therapeutic protocol with DAAs. One patient that presented RAS (V321I) was classified as non-responder, and combination of RAS C316N and Q309R does not necessarily imply in resistance to treatment in this cohort of patients. Data presented herein highlights the relevance of studying circulating variants for a better understanding of HCV variability and resistance to the therapy. Furthermore, the feasibility of carrying out genotyping and RAS phenotyping analysis by using DSS card for the potential of informing future treatment interventions could be relevant to overcome the limitations of processing samples in several location worldwide, especially in LMICs.

## Introduction

Hepatitis C virus (HCV) is a major cause of chronic hepatitis and hepatocellular carcinoma globally [1]. This pathogen has infected nearly 180 million people worldwide [2, 3], and about 80 % of infections are reported in Low and Middle-income countries (LMICs) [4, 5]. Despite this high prevalence rate, data on molecular epidemiology of HCV seem relatively scarce in LMICs, which can be associated with a lack of adequate throughput facilities to study the virus [6, 7].

HCV is an enveloped, icosahedral, positive sense single-stranded RNA virus that belongs to the family Flaviviridae, genus *Hepacivirus* [8, 9]. The viral genome is composed of approximately 9600 nucleotides and represents an uninterrupted open reading frame (ORF) encoding a polyprotein precursor of approximately 3000 amino acids [10, 11]. The HCV polyprotein is co- and post-translationally processed by a combination of cellular and viral proteases into three structural proteins (Core, E1, and E2) and seven non-structural (NS) proteins (P7, NS2, NS3, NS4A, NS4B, NS5A, and NS5B). Phylogenetic analysis of global strain diversity resulted in the distinction of eight HCV genotypes (1 to 8) and 86 subtypes (a, b, c etc.), based on the nucleotide sequences of the Core/E1 and NS5B regions [3, 10, 12–16].

The NS5B is the viral RNA-dependent RNA-polymerase (RdRp) responsible for HCV RNA replication [15], an enzyme that lacks proof-reading activity, leading to the emergence of intrahost diversity of viral populations circulating in the blood of an individual [10]. The variability and dynamic of viral infection in the host organism are related to the progression of antiviral treatments, which can result in selective pressure for the emergence and spread of drug-resistant viral strains [10, 17]. The initial therapies used against HCV were interferons-based (IFN), resulting in several collateral effects. In 2015, Food and Drug Administration (FDA) approved the therapy with IFN-free direct-acting antivirals (DAAs) [12, 18], classified as NS3/4A protease inhibitors, NS5A inhibitors and NS5B polymerase inhibitors (NI - nucleoside inhibitor or NNI - non-nucleoside inhibitor). Treatment with DAAs presents high levels of sustained virologic response (SVR) rates, typically >90 %, contributing to both the control of HCV epidemics and improvement of the quality of life of patients, with reduced side effects compared to IFN [12]. However, under treatment, resistance-associated substitutions (RAS) might occur into the genomic regions related to the viral proteins targeted by anti-HCV drugs, which may confer resistance to the DAA classes [12, 19].

The three-dimensional structure of the viral NS5B polymerase contains three domains: Palm, Fingers, and Thumb [20–22]. Structural analyses of this protein and its active site in the palm domain demonstrated that substitutions that occurred close to the catalytic triad of HCV NS5B polymerase interfered with the effectiveness of antivirals. The most common RAS that affects the activity of NS5B inhibitors are L159F, D244N, S282T/R, Q309R, D310N, and A333E [23–25].

Surveying the viral evolution and identifying RAS in the NS5B of HCV is a relevant approach. However, HCV genotyping and RAS diagnosis can be a challenge in many locations, as for instance in Brazil. In this context, the use of desiccated patient specimens such as dried serum spots (DSS) or dried blood spots (DBS) may facilitate the monitoring of HCV infections in regions with limited access to laboratory services, reaching vulnerable populations [26–29]. DBS samples blotted on filter paper have revolutionized the point of care management of patients living with HCV and the study of HCV in developed countries [30, 31]. DSS requires traditional phlebotomy prior to blotting but provides identical downstream storage and transport potential where primary diagnostic facilities and / or secondary reference laboratories have significant distance from point of care [32].

Regrettably, LMICs that should benefit more from using this technology are lagging far behind with relatively few studies utilising the technique published [33, 34]. Besides, the HCV ribonucleic acid degradation of the specimen (whole blood/serum/plasma) at the collection site poses a serious challenge in LMICs, especially when serology or molecular studies cannot be performed immediately, or additional analyses requires further shipment to a reference laboratory. The dried spot technique proffers solutions to this problem. More importantly, many studies have shown no significant variation in the assay performance between the DBS and direct sample methods [31, 35, 36]. Also, DBS been proven efficacious for screening high-risk patients such as intravenous drugs users [37, 38] and require no cold chain for onward transportation from the sample collection site to the point of final analysis [39, 40]. Although, to date there is no standard operating procedure for elution and recovery of HCV RNA from desiccated material [41], the World Health Organization (WHO) testing guidelines recommend the use of DBS specimens as an option for HCV testing and analysis, especially where there are no facilities for samples processing [42].

In this study, we utilised surplus archived serum taken for routine HCV diagnostics to prepare DSS cards to carry out additional genotyping and RAS identification by analysing the NS5B palm domain variability and treatment response data in a cohort of chronically infected patients from São Paulo state, Brazil. Considering that Brazil has limited facilities for genotyping and resistance testing, our data provides relevant information to overcome the limitations of processing samples in several location worldwide, especially in the LMICs [43].

## Methods

### Clinical samples

**Fig. 1.**
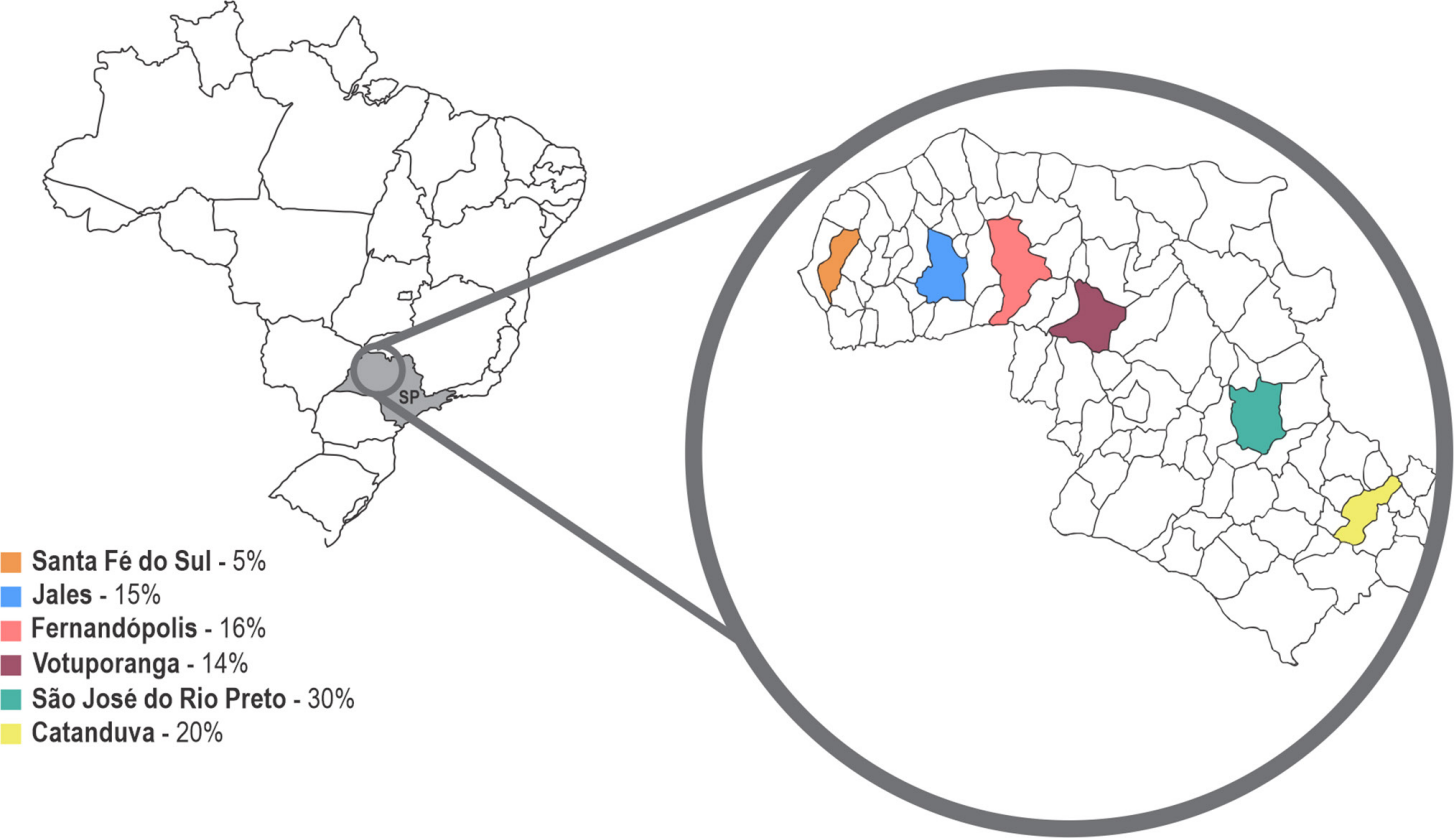
Location of the samples collection distributed by percentage according to the city in the highlighted northwest region of the state of São Paulo (SP).

**Fig. 2.**
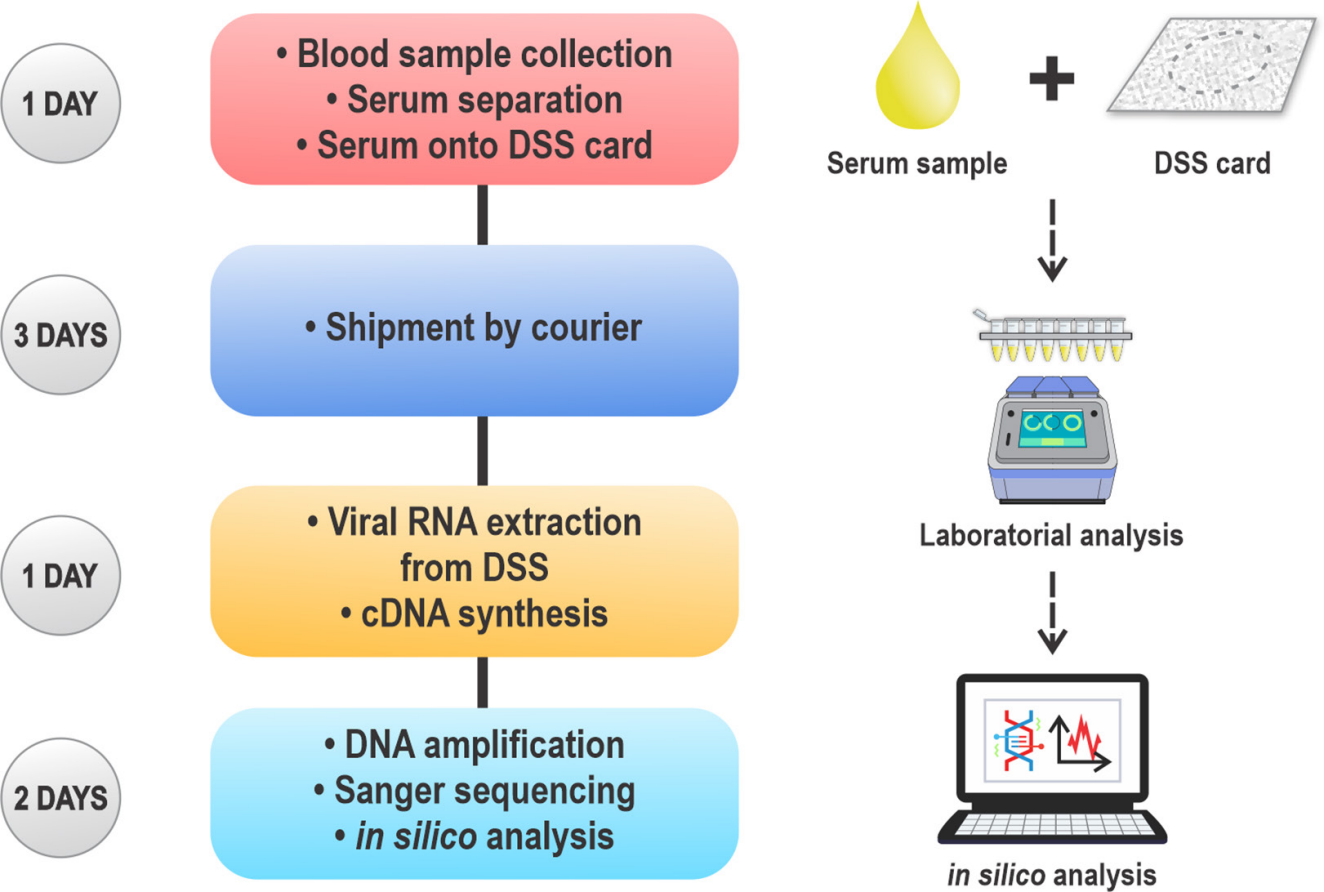
Remote genotyping workflow: duration between conveyed samples by dried serum spots (DSS) card until final results.

Eighty serum samples from patients previously diagnosed positive for HCV attending four specialized HCV screening and treatment centres from 2010 to 2017 were obtained after long-term storage at −20 °C or below. Samples were collected in specialized centres of the northwest region of the state of São Paulo, Brazil, serving the cities of São José do Rio Preto, Catanduva, Votuporanga, Fernandópolis, Jales, and Santa Fé do Sul (Fig. 1). Samples were taken for routine diagnostic investigation according to the varied methods and storage protocols of the four centres. The dried serum spot samples that were RNA positive presented a viral load ranging from 3.0×10^4^ IU ml^−1^ to 5.3×10^7^ IU ml^−1^. The core demographic data such as age, sex, viral loads, serum collection centres, and suspected route of infection were analysed. Additional retrospective data analysis as outlined in the following three sections of this study were completed within approximately 1 week of sample receipt (Fig. 2).

### Dried serum spot preparation and HCV RNA elution

Thirty microlitres of each serum sample was spotted onto a Protein Saver 903 Card (GE Healthcare), filling the demarcated 12 mm diameter circles and allowed to air dry at room temperature (25 °C) for approximately 1 h, then stored at 4 °C prior to shipment. Ambient shipment of the samples took approximately 1 week by international courier (DHL). Each entire serum spot was recovered from the DSS Card, by using a 5 mm puncher to take multiple discs from the sample area. The discs were suspended in a 2 ml tube containing 300 µl Phosphate Buffered Saline (PBS). The tubes were incubated with agitation at room temperature for 10 min, and then 140 µl of supernatant virus suspension/eluate were obtained for RNA extraction. Single punches of clean card were taken between samples and negative extraction controls undertaken to counteract and monitor for carryover contamination.

### NS5B amplification and sequencing

RNA extraction was carried out using QIAamp MinElute virus spin RNA extraction kit (QIAGEN) according to the manufacturer’s instructions. Then, 50 µl HCV RNA eluate from each sample were stored at −70 °C and used for cDNA synthesis. A 20 µl RNA extract (a template) was added to a lyophilized EcoDry Premix (random hexamers) (Takara-Clontech), incubated at 42 °C for 60 min to generate complementary DNA, in a reverse transcriptase-polymerase chain reaction.

**Fig. 3.**
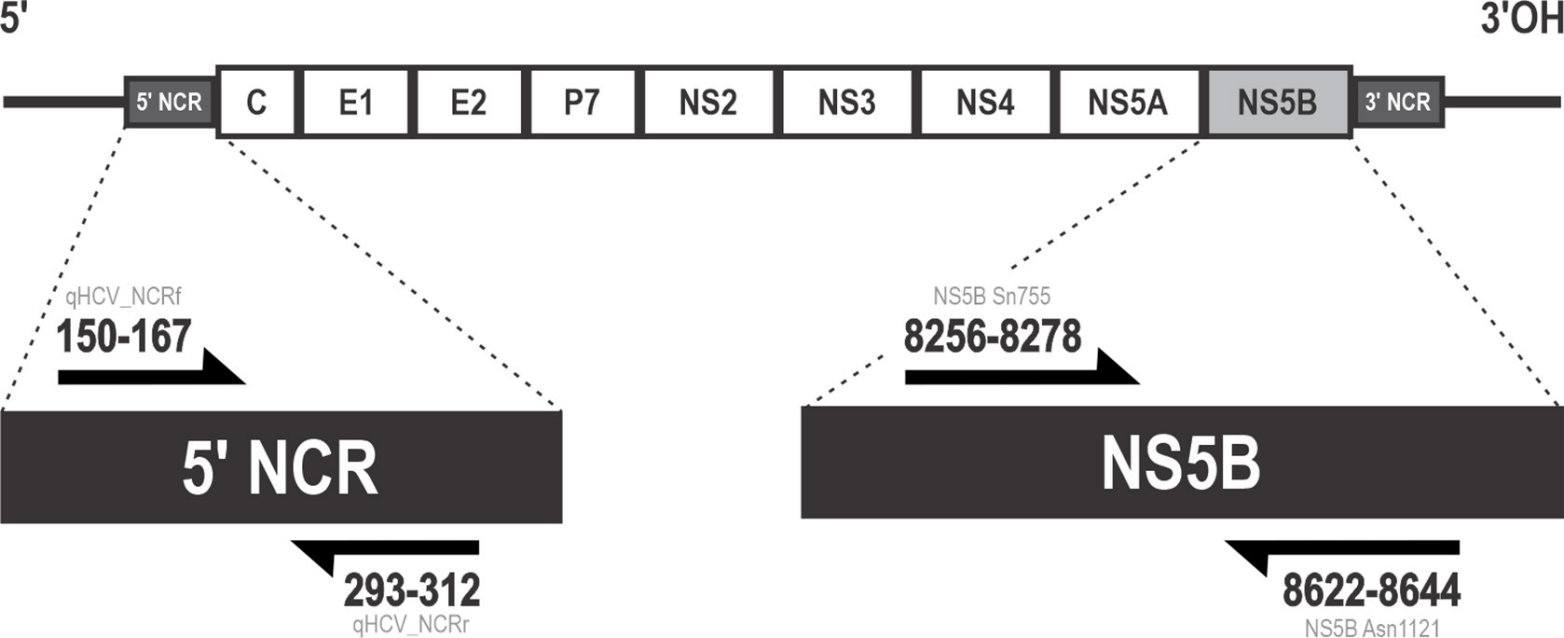
Schematic representation of the HCV genome, its polyprotein and the primers used in the fragment amplification.

PCR reactions were performed using HotStarTaq (QIAGEN), according to manufacturer's instruction. Primers qHCV_NCRf (5′-GCGCAACCGGTGAGTACA-3′) and qHCV_NCRr (5′-ACTCGCAAGCACCCTATCAG-3′) [44] were used to amplify the 5′ untranslated region; and Sn755 (5′-TATGAYACCCGCTGYTTTGACTC-3′) and Asn1121 (5′-GCNGARTAYCTVGTCATAGCCTC-3′) [45] for NS5B (Fig. 3). Reactions were performed using the following conditions: 94 °C for 15 min for enzyme activation; 55 cycles of denaturation at 95 °C for 20 s, annealing at 55 °C for 20 s and extension at 72 °C for 20 s; and final extension at 72 °C for 10 min. Expected amplicon was 388 bp, confirmed by electrophoresis. PCR products were diluted 1:10 with water and ambient shipped direct for Sanger sequencing (Source BioScience, Nottingham, UK).

### Sequence analysis, RAS identification and phylogeny reconstruction

Putative Sanger sequences were visualized and manually edited by removing the terminal primer sequences using Finch TV software. Sequence assembly, alignment, and phylogenetic analysis were performed using mega six software [46]. Sequences were trimmed to 288 bases in length corresponding to 8311–8598 of H77 reference, accession NC_004102. The curated NS5B sequences were uploaded to seven different online HCV subtyping tools to compare outcomes: [1] Los Alamos database (LANL) [47]; [2] Virus Pathogen Database and Analysis (VIRP) [48]; [3] Oxford HCV automated subtyping tool (version 2.0) [49]; [4] NCBI genotyping tool [50]; [5] Geno2Pheno HCV Subtyping tool [51]; [6] HCV Comet [52]; and [7] HCV Glue Subtyping tool [53]. Further characterization of the sequences was performed by phylogenetic analysis using each confirmed HCV genotypes/subtypes for easy visualization/visibility from reference datasets in the International Committee on Taxonomy of Viruses (ICTV) to determine HCV subtype [15].

Maximum-likelihood phylogeny reconstruction with bootstrap evaluation was conducted in mega X programme, using the GTR nucleotide substitution model and gamma (Γ) distribution of rate variability among sites (GTR + gamma) [54]. The bootstrap (1000 replicates) cut-off greater than or equal to 70 % of each cluster clades was considered a reliable confidence threshold [55].

Amino acid sequence data corresponding to amino acid positions −223 to −339 were aligned using the Geno2Pheno software platform and NS5B RAS was thereby determined. The investigated RAS were selected based on the substitutions described in the literature in the NS5B palm domain genomic region (Table 1). Study sequences were submitted to GenBank, with accession numbers OL752714 - OL752779.

**Table 1.** Substitutions in the NS5B polymerase palm region related to resistance to direct-acting antivirals

RAS	Genotype	Reference
L 159 F	1a, 1b, 3a	Donaldson *et al*. (2014); Svarovskaia *et al*. (2014); Tong *et al*. (2014); Lontok *et al*. (2015); Noble *et al*. (2017) [23, 25, 103–105]
D 244 N	3a	Asahina *et al*. (2005); Castilho *et al*. (2011) [24, 96]
S 282 T/R	1a, 1b	Donaldson *et al*. (2014); Tong *et al*. (2014); Svarovskaia *et al*. (2016) [23, 104, 106]
Q 309 R	1a, 1b, 3a	Hamano *et al*. (2005); Castilho *et al*. (2011) [24, 107]
D 310 N	1b, 3a	Asahina *et al*. (2005); Castilho *et al*. (2011) [24, 96]
C 316 H/N/Y	1a, 1b	Shi *et al*. (2008), McCown *et al*. (2009); Castilho *et al*. (2011); Donaldson *et al*. (2014); Lontok *et al*. (2015); Noble *et al*. (2017) [23–25, 105, 108, 109]
L 320 F	1a, 1b, 3a	Tong *et al*. (2014); Svarovskaia *et al*. (2016) [104, 106]
V 321 A/I	1b, 3a	Donaldson *et al*. (2014); Svarovskaia *et al*. (2014); Lontok *et al*. (2015); Noble *et al*. (2017); Hezode *et al*. (2018) [25, 103, 105]
A 333 E	1a	Hamano *et al*. (2005); Castilho *et al*. (2011) [24, 107]

### Statistical analysis

All the data were analysed appropriately by GraphPad Prism version 8.0.1 for Windows, GraphPad Software, San Diego, California USA (www.graphpad.com). The descriptive data are presented as mean±standard deviation. The data were categorized in groups and analysed according to log normality test. Non-parametric data were compared by the Mann Whitney test, and the parametric data, by the *t*-test. Statistical inferences were based on a *p*-value of less than 0.05 as significant.

## Results

### Clinical characteristics of the patients

The study included 20 % of samples from Catanduva, 16 % from Fernandópolis, 15 % from Jales, 30 % from São José Do Rio Preto, 14 % from Votuporanga and 5 % from Santa Fé Sul (Fig. 1). The participants were within the age ranging from 30 to 80 years. Males were predominant, accounting for 53 %. The frequency of suspected route of infections was 33 % parenteral /drug use, 12 % of blood transfusion, 12 % surgical/tattoo, 11 % sexual contact, whilst 32 % did not disclose any known route of infection. Two patients were HIV/HBV co-infected, while ten tested positive for HIV infection (Table 2).

**Table 2.** Demographic characteristics of the study samples

Characteristics	no. (%)	GT 1a (*N*=34)	GT 1b (*N*=18)	GT 3a (*N*=12)	GT 2a (*N*=1)	GT 2c (*N*=1)	*P*-value
**Gender**							
Male	35 (53)	18	9	6	1	1	0.757
Female	31 (47)	16	9	6	–	–	
**Age Group**							
<39	10 (15)	5	3	2	–	–	0.042
40–49	21 (32)	14	3	4	–	–	
50–59	15 (3)	8	4	3	–	–	
60–69	14 (21)	5	7	2	–	–	
70–79	5 (8)	1	1	1	1	1	
80–89	1 (1)	1	–	–	–	–	
**Suspected route of infection**							
Blood products	8 (12)	4	3	1	–	–	0.657
Drug use/parenteral	22 (33)	12	5	4	–	1	
Sexual contact	7 (11)	3	2	2	–	–	
Surgical/tattoo	8 (12)	3	1	4	–	–	
Unknown	21 (32)	12	7	1	–	–	
**Viral Load**							
<6x10^4^	1 (1)	–	1	–	–	–	0.801
6×10^4^ - 8×10^4^	2 (3)	2	–	–	–	–	
>8x10^4^	63 (96)	32	17	12	1	1	
**Co-infection**							
HIV	10 (15)	6	3	1	–	–	0.977
HIV/HBV	2 (3)	2	–	–	–	–	
Unknown	6 (9)	23	14	9	1	1	

Concerning the treatment profile of the patients, 23 % of the analysed samples were from naive patients, which means they had not undergone previous treatment against HCV. Patients who undergone therapy with direct-action antivirals (DAAs) presented three distinct patterns of response to treatment, classified as: i) the sustained virological response (SVR), meaning that blood tests of the patient continue to show no detectable RNA 12 weeks or more after treatment; ii) end-of-treatment responders (ETR), when there is no detectable HCV RNA in the blood at the completion of treatment but it was detected after the end of the treatment; iii) or non-responders (NR), when HCV RNA is detectable during and after the treatment. In total, 90 % of the patients were previously treated with interferon and ribavirin, and 58 % of them submitted to a second protocol of treatment with DAAs. Among patients who used sofosbuvir and daclatasvir, or simeprevir, as the second therapeutic approach, 75 % did not respond to treatment and 25 % were ETR, representing 0 % of SVR. Therefore, therapy with DAAs followed by a previous IFN-based protocol demonstrated reduced SVR. Patients who received DAAs as the first therapeutic approach represented 39 % of the patients, with an SVR rate of 96 % (Table S1, available in the online version of this article).

### DSS samples processing and genotype assignment

**Fig. 4.**
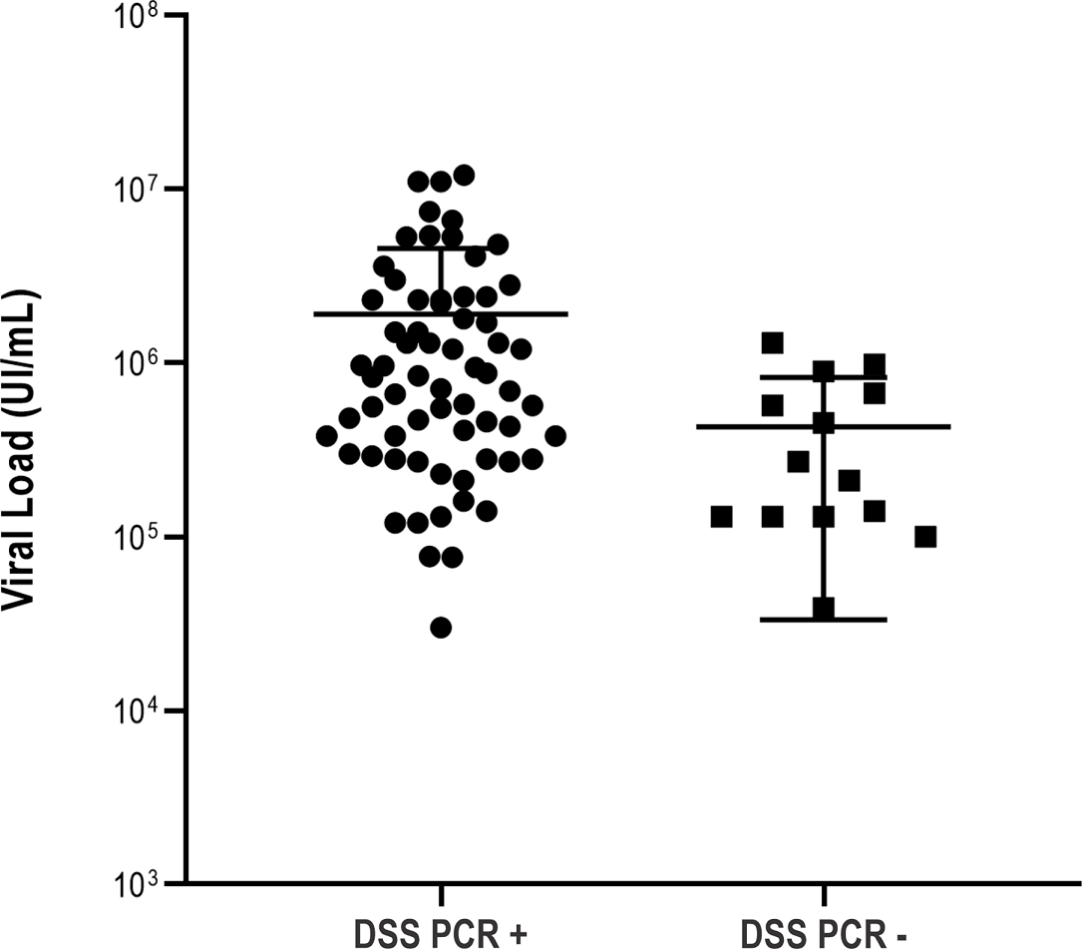
RNA viral load in PCR positive and PCR-negative Brazilian HCV patients; displaying the impact of viral load on PCR outcome

From the 80 DSS samples investigated in this study, 66 (83 %) partial sequences of the NS5B coding region corresponding to the NS5B palm domain were successfully amplified and sequenced for analysis. A possible reason for the failure of 17 % of unamplified samples despite medium to high viral loads (3.04×10^4^ IU ml^−1^ to 5.39×10^7^ IU ml^−1^) in the initial diagnostic assay from the same specimen might be related to inconsistent handling over a broad collection period at the various collection sites. Each diagnostic and long-term storage pathway were prior to our retrospective investigation and thus may have been sub-optimal in some instances, but no further information was available. There was statistical difference in the diagnostic lab-tested HCV RNA viral load between the DSS PCR positive and negative samples (*P*=0.0042) (Fig. 4).

**Fig. 5.**
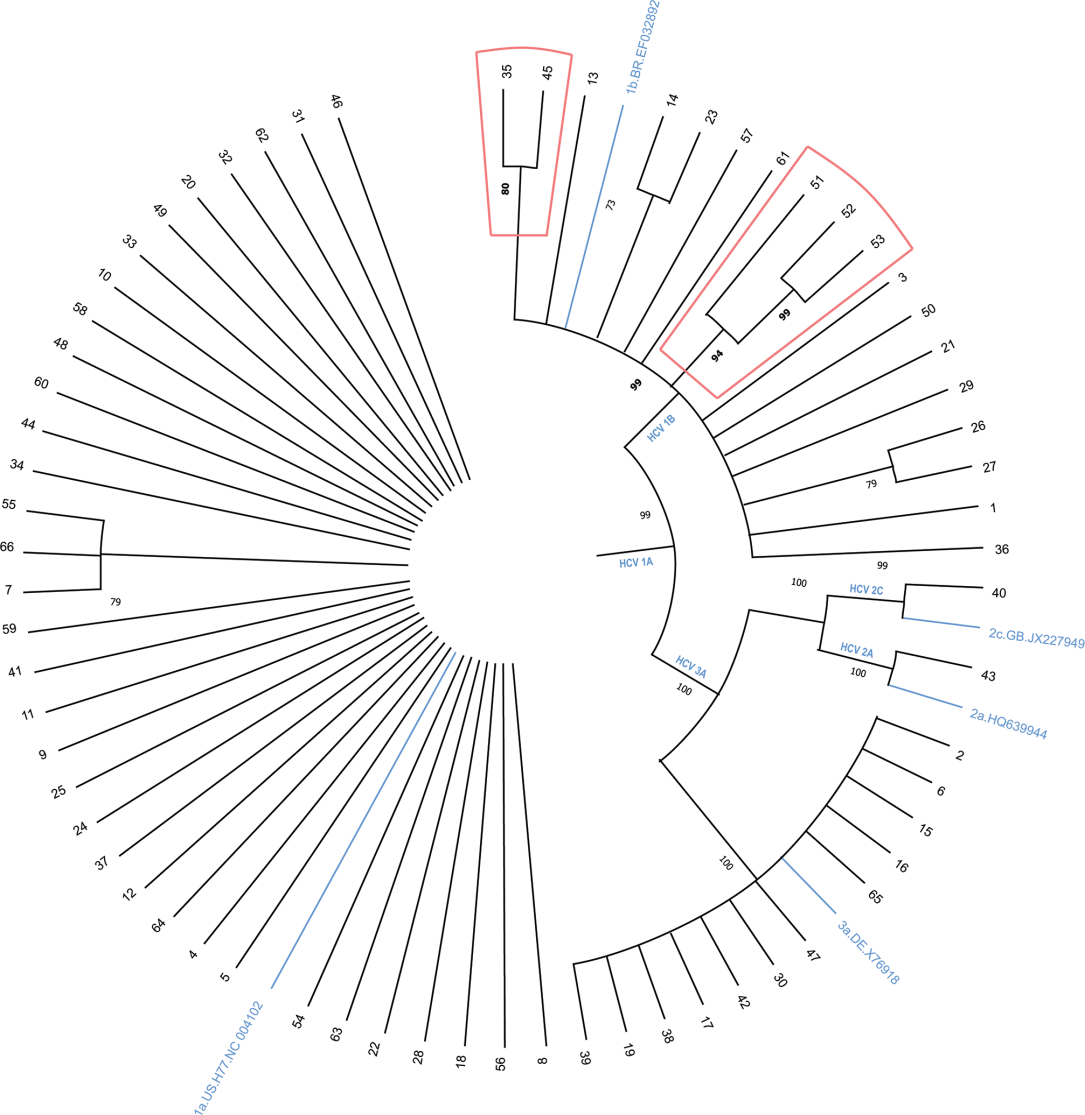
Phylogenetic tree not rooted with 66 sequences of 308 nucleotides from the NS5B region from samples from patients infected with HCV genotypes 1a, 1b, 2a, 2c and 3a. Red highlights indicate the clade of sequences 51, 52, and 53, which contains RAS C316N and Q309R, and the clade of sequences 35 and 45, with V321I. Bootstrap values obtained with 1000 replicates. The figure shows the bootstraps with values above 60.

Genotype assignment demonstrated 100 % concordance using all of the seven online subtyping tools. The online tool prediction was also consistent with the phylogenetic analysis (Table S2). More importantly, all the sequences could be assigned at the subtype level (Fig. 5). The most frequent genotypes were 1a (34/66, 52%), 1b (18/66, 27%), and 3a (12/66, 18%), with the remainder being subtypes 2a (1/66, 1.5%) and 2c (1/66, 1.5%). There were no significant associations between subtypes/gender (*P*=0.757) and subtypes/suspected route of infections (*P*=0.765). However, the association between subtypes and age groups was significant (*P*=0.0442). Genotype 1a has the highest frequency in the age groups of 40–49 (41 %, 14/34) and 50–59 (24 %, 8/34). Genotype 1b was predominant in the age group 60–69 years, with a frequency of 39 % (7/18). Genotype 3a has the lowest incidence when compared to the others, yet was the second predominant in 40–49 group, with 33 % (4/12) frequency (Table 2).

### Resistance association prediction

The palm domain of NS5B (aa 188 to 227 and aa 287 to 370) [56] was the region evaluated for RAS investigation by subjecting the sequences to the Geno2Pheno software tool. The amino acid sequence in this analysis overlaps 240–342 fragment. This segment is well characterized and was used for the analysis (Table 1). Either RAS C316Y and Q309R, were observed in the sequences of patients infected with HCV 1a (Table 3). Among genotype 1b sequences, samples of patients 51, 52 and 53 presented RAS Q309R and C316N, previously described for the amplified region (Table 1); and samples of patients 35 and 45 presented RAS V321I (Table 3).

**Table 3.** NS5B Resistance associated substitution (RAS) analysis. NS5B sequences generated for 66 samples were analysed with Geno2Pheno software for the presence of described RAS by genotype to DAAs

Genotype	No. of patients	Patients with RAS	Amino acid position associated with NS5B RAS								
			L 159 F	D 244 N	S 282 T/R	Q 309 R	D 310 N	C 316 H/N/Y	L 320 F	V 321 A/I	A 333 E
1a	35	16				15		1 C 316 Y			
1b	17	5				3		3 C 316 N		2 – V 321 I	
2a	1	0									
2c	1	0									
3a	12	0									

### Phylogenetic analysis

The tree generated from the phylogenetic reconstruction with the 66 analysed sequences is shown in Fig. 5. Genotypes 1a, 1b, 2a, 2c and 3a were grouped in different monophyletic branches, strongly sustained by the bootstrap values of 99, 99, 100, 99, and 100%, respectively, confirming previous genotyping analyses (Fig. 5). HCV 1b sequences 51, 52 and 53, which presented RAS C316N and Q309R, grouped in the same monophyletic branch, with 94 % of bootstrap. Additionally, sequences 52 and 53 grouped in a clade strongly sustained by a bootstrap of 99 % (Fig. 5). Furthermore, HCV 1b sequences 35 and 45 presented RAS V321I, and grouped in a clade sustained by the bootstrap value of 80 %.

## Discussion

In this study, we reported an 83 % (66/80) success rate using dried serum spots (DSS) cards to genotype samples of HCV patients from the northeast region of São Paulo, Brazil. This is comparable to the 85 % success rate reported by Soulier and colleagues*,* in which they determined the HCV genotype using a similar DBS technique [57]. Available evidence showed that HCV RNA in DBS can be detected at levels >1000 IU ml^−1^ and detection limit range between 178 to 1779 IU ml^−1^ [58]. In accordance to the recent WHO guidelines on Hepatitis B and C testing, a detection limit of 3000 IU ml^−1^ or lower is acceptable and would identify 95 % of those with viraemic infection [59, 60]. Also, this limit is higher than the 45 IU ml^−1^ detection limit for the plasma sample [61]. This method has shown immense benefit in screening, surveillance, diagnosis, and monitoring of HCV treatment [57, 62], aside from success rates of about 90–100 % reported by Mahajan and co-workers and Vázquez-Morón and colleagues [41, 63].

Whilst we utilised Sanger sequencing, Next-generation Sequencing (NGS) technology could equally be applied to nucleic acid extracted from DSS cards. We have previously shown comparable results between Sanger and Nanopore sequencing technologies with Hepatitis B amplicons from DSS-derived material, but improved depth of coverage of minority variants from the NGS reads [32]. Elsewhere, Alidjinou and co-workers have evaluated Sanger sequencing with alternative NGS technology for HIV-1 drug resistance testing in treatment-naive patients, with NGS again facilitating detection of additional minority drug-resistant mutations [64].

According to our data, genotypes 1a, 1b, and 3a were the most prevalent, followed by 2a and 2c, respectively. It confirms the worldwide panorama since the widespread HCV genotypes are 1 (44 %) and genotype 3 (25 %) [65]. The prevalence of HCV genotype distribution in this study is also in accordance with previous studies performed in Brazil [66–68]. Recently, Mutini and colleagues described a high prevalence of genotypes 1a (41%), 1b (30%), 3a (24%), and 2 (4 %) in about 29071 of either healthy people or blood donors in Brazil. Our data is in agreement with most of previous studies in São Paulo state; the predominant genotypes being 1a, 1b, and 3a [69, 70]. However, genotypes 3 and 1b have been reported as the most prevalent genotypes in Salvador [71] and Pernambuco [67], respectively; both in north-eastern Brazil.

Herein, the suspected acquisition of HCV infection was 33%, similar to previous studies that observed 36 % of risk of transmission by parenteral/drug use in the North and Northeast regions in Brazil [72, 73]. Coutinho and co-workers, however, emphasize this transmission route as the most significant in the country. According to their data, the odds of HCV infection are 85 % higher among multidrug users and almost eight times higher among injecting drug users [74].

No significant association was observed between genotypes and suspected route of infection, despite the majority of patients being infected with subtypes 1a through parenteral/injection drug use. This is in agreement with some studies which have associated genotype 1a with injectable drugs users [75, 76]. However, some studies in developed countries have shown that genotype 3a is more prevalent in this group of HCV infected patients [77]. These different profiles may likely be due to some particular variables [78]. There was a significant association between subtypes and age group, being genotype 1a the most prevalent in the age group 40–49 years. This data corroborates with the study of Guimarães and co-workers that underpins the idea of late diagnosis of chronic HCV infection and thus a historical predominance of genotype 1a [79]. The risk of HCV transmission via blood transfusion (12 %) in this study is low compared to previous study (38%) [80]. This is expected because of high transfusion-transmitted disease awareness and the level of blood donor screening [81–83]. Also, the risk of surgical/tattooing agrees with previous reports of negligence to the sterilization of body piercing instruments in Brazil [84]. Sexual exposure risk is 11 % in this study; the transmission of HCV via sex is controversial in HCV infection [85, 86]. However, studies have shown high risk in people that trade sexual services, injecting drugs, STI – coinfection (Sexually Transmitted Co-Infections) and, MSM (men who have sex with men) [74, 87, 88]. The HCV co-infection prevalence observed herein is consistent with the previous reports in Brazil [89].

The new findings on HCV viral replication allowed a better understanding of non-structural 5B (NS5B) activity, resulting in the polymerase inhibitors implementation. The DAAs of this class act mainly in the NS5B palm domain, between the amino acids at positions 287 and 371, which is the most well-conserved domain and contains the catalytic residues. Therefore, RAS to these antivirals are concentrated in this HCV region [23]. Until now, there are few studies in Brazil associating the effectiveness of DAA therapeutic regimens and the role of RAS in a flaw treatment outcome [90]. Monitoring the identification and circulation of the variants in Brazilian population, and worldwide, is relevant to evaluate the possible impacts in therapeutic failure with DAAs [91]. One reason for the lack of data on the circulation of RAS in several countries is the difficulty in testing HCV infected patients [92, 93]. The DSS sampling is, however, a reliable alternative specimen collection method that may facilitate the monitoring of ongoing RAS into vulnerable populations [26–29].

As stated in our data, patients who previously undergone therapy with interferon (INF) and ribavirin (RBV) were submitted to a second therapeutic approach, using DAAs (sofosbuvir and daclatasvir, or simeprevir). These patients did not achieve SVR, suggesting that previous therapy with INF and RBV could interfere in the effectiveness of subsequent therapy with DAAs. Additionally, patients who received DAAs as the first therapeutic approach presented higher SVR rates. Treatments with INF and RBV present many side effects and, the RBV mechanism action is still unclear. It is known that RBV is a purine analogue, and it is involved in several cellular pathways, acting synergistically with INF [94]. An important antiviral mechanism of action of RBV is the inhibition of HCV replication, which interferes with both the NS5B polymerase and the NS5A multifunctional protein [95]. The action of RBV as a purine analogue, associated with the lack of corrective activity of NS5B, results in high rates of mutations in the viral variants of chronically infected patients undergoing this therapeutic protocol [96]. It could potentially cause the failure of the first treatment and result in a bottleneck effect in the viral population. In that way, previous treatment with INF and RBV could select resistant variants, which could explain why patients did not respond to the treatment with DAAs as a second therapeutic approach. Jardim and co-workers evaluated intrahost viral diversity in HCV chronically infected patients and suggested that the composition of quasispecies population at the beginning of the treatment, followed by an increase in some predominant quasispecies after treatment of NR and ETR in their patients, represented an advantage for the virus as it remains in the organism [97]. Although the heterogeneous population of HCV sequences *in vivo* is the main cause of DAAs treatment failures, it seems to have no differences between the hepatic and plasma viral quasispecies, as demonstrated by Hedegaard and colleagues [98]. In this context, based in preclinical *in vivo* evidence, an alternative presented by Vercauteren and colleagues was that the addition of an entry inhibitor to an anti-HCV DAA regimen restricts the breakthrough of DAA-resistant viruses. According to their work, this strategy may prevent therapeutic failure caused by RAS developing, and this combination can increase response rates, especially in difficult-to-treat patient populations [99].

Treatment resistance is associated with several factors. The resistance mechanism associated with RAS is not completely understood; therefore the presence of an isolated substitution may not confer resistance to the treatment. It is known that the combination of RAS L159F and L320F or C316N, for example, directly interferes in the action of sofosbuvir, targeting the palm domain of NS5B [23, 100]. Furthermore, a study by Uchida and colleagues demonstrated that patients with previous treatment with RBV had a lower frequency of C316N replacement than those who did not previously treat with this drug [101]. The RAS C316N / Y, Q309R and V321I are also associated with DAA resistant variants. The spread of these mutations in the northwest region of São Paulo, Brazil, is worrisome, as it may be associated with an increase in the number of individuals resistant to the available treatment. Therefore, surveillance of circulating variants in the infected population should be performed regularly. A recent study evaluated more than 100 HCV-infected patients in Rio de Janeiro, Brazil, identifying amino acid substitutions in positions 159 and 316 of the NS5B of genotype 1b [91]. Although the majority of Brazilian patients with HCV are susceptible to therapeutic regimens with DAAs, the presence of these RAS in the variants of this region opens avenues for the implementation of resistant viral variants [91].

Here both RAS C316N and Q309R located in the NS5B palm region were observed in the sequences from patients 51, 52, and 53. These sequences were grouped in the same monophyletic branch, showing high similarity among the variants of these patients. Despite these patients presented SVR after therapy with DAAs, the identification of the RAS in these samples is a relevant indicator that these substitutions are circulating in this location. Mutations in the amino acid C316 have been frequent in Asian variants, and have also been reported in many analyses in Brazil [102]. Moreover, a second monophyletic branch was presented in the reconstruction of the topology of the phylogenetic tree, grouping sequences 52 and 53, demonstrating that these sequencers are genetically closer than to sequence 51. Interestingly, patients 52 and 53 were assessed in Votuporanga, unlike patient 51 who was examined in São José do Rio Preto. Besides that, the RAS V321I was observed in sequences 35 and 45, which grouped in a separated clade. One of the sequences is from a patient from Jales, the only non-responder patient, and presented one of the targets RAS we listed in the studied population.

To the best of our knowledge, this study has shown for the first time that cascade of HCV care such as diagnosis, genotyping, and drug resistance analysis is achievable using DSS. This can be implemented in LMICs where facilities are limited. Finally, it plays an important role in the accomplishment of the WHO mandate towards global eradication of HCV by the year 2023.

## Supplementary Data

Supplementary material 1
